# Controversies in Podocyte Loss: Death or Detachment?

**DOI:** 10.3389/fcell.2021.771931

**Published:** 2021-11-22

**Authors:** Lijun Yin, Lu Yu, John Cijiang He, Anqun Chen

**Affiliations:** ^1^ Hunan Key Laboratory of Kidney Disease and Blood Purification, Department of Nephrology, Institute of Nephrology, The Second Xiangya Hospital at Central South University, Changsha, China; ^2^ Department of Health Sciences, Boston University College of Health and Rehabilitation Sciences: Sargent College, Boston University, Boston, MA, United States; ^3^ Division of Nephrology, Department of Medicine, Icahn School of Medicine at Mount Sinai, New York, NY, United States; ^4^ Renal Program, James J. Peters Veterans Affairs Medical Center at Bronx, New York, NY, United States

**Keywords:** podocyte loss, cell death, detachment, apoptosis, autophagic cell death, mitotic catastrophe, anoikis, pyroptosis

## Abstract

Glomerular podocytes are characterized by terminally differentiated epithelial cells with limited proliferating ability; thus, podocyte loss could not be fully compensated by podocyte regeneration. A large body of clinical studies collectively demonstrated that podocyte loss correlated with glomerular diseases progression. Both podocyte death and podocyte detachment lead to podocyte loss; however, which one is the main cause remains controversial. Up to date, multiple mechanisms are involved in podocyte death, including programmed apoptotic cell death (apoptosis and anoikis), programmed nonapoptotic cell death (autophagy, entosis, and podoptosis), immune-related cell death (pyroptosis), and other types of cell death (necroptosis and mitotic catastrophe-related cell death). Apoptosis is considered a common mechanism of podocyte loss; however, most of the data were generated *in vitro* and the evidence of *in vivo* podocyte apoptosis is limited. The isolation of podocytes in the urine and subsequent culture of urinary podocytes *in vitro* suggest that detachment of viable podocytes could be another important mechanism for podocyte loss. In this review, we summarize recent advances that address this controversial topic on the specific circumstances of podocyte loss.

## Introduction

**FIGURE 1:**
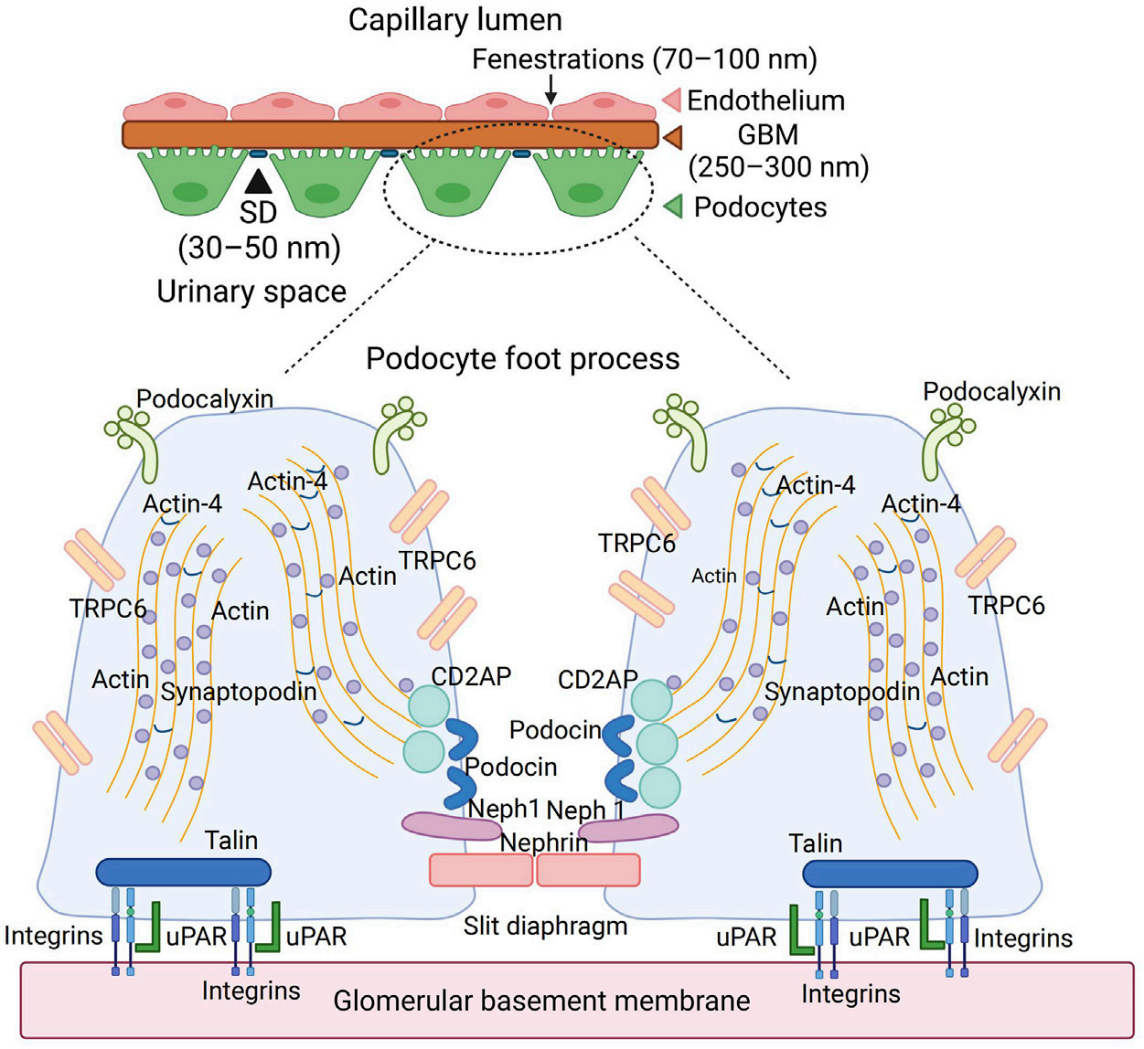
The anatomy of glomerular basement membrane and podocyte. Podocyte is the last barrier of the glomerulus filtration membrane, which contains a slit diaphragm with 30–50 nm width. The abundant cytoskeleton proteins, such as actin and synaptopodin, maintain the normal shape of podocytes. The membrane proteins, such as podocalyxin, TRPC6, podocin, and nephrin, played an essential role in the podocyte function. Podocyte anchored into the GBM through integrins and uPAR interaction. TRPC6, transient receptor potential cation channel subfamily C member 6; GBM, glomerular basement membrane; uPAR, urokinase-type plasminogen activator receptor; CD2AP, CD2-associated protein.

Glomerular podocytes account for approximately 30% of glomerular cells in number, which constitute glomerular filtration barrier (GFB) together with endothelial cell and glomerular basement membrane (GBM) (Nishad et al., 2021). Podocyte is the last barrier of GFB (Figure 1), the injury of which is a key manifestation of glomerular diseases, including minimal change disease (MCD), focal segmental glomerulosclerosis (FSGS), and diabetic nephropathy. Podocytes are characterized by terminally differentiated epithelial cells with a limited capacity of proliferation; thus, podocyte loss cannot be fully compensated by regeneration from nearby healthy podocytes. One of the features of glomerulosclerosis is the continuous loss of podocytes, which correlates with the progression of glomerular diseases (Lemley et al., 2002). When the loss of podocytes per glomerulus is above 20%, glomerulosclerosis takes place. When the podocyte loss is more than 40%, reduced renal function occurs (Wharram et al., 2005). However, the underlying mechanism of this remains unclear.

Podocyte loss can be caused by either podocyte death or podocyte detachment; however, which one is the main cause remain controversial. The major pathways involved in podocyte death include programmed apoptotic cell death (apoptosis and anoikis), programmed nonapoptotic cell death (autophagy, entosis, and podoptosis), immune-related cell death (pyroptosis), and other types of cell death (necroptosis and mitotic catastrophe-related cell death) (Galluzzi et al., 2018). Apoptosis is a common mechanism of podocyte loss; however, most of the data were generated *in vitro* and the *in vivo* evidence of podocyte apoptosis is limited. The isolation of podocytes in the urine and subsequent culture of urinary podocytes *in vitro* suggest that detachment of viable podocytes could be another important mechanism for podocyte loss. In this review, we will summarize recent advances that address the mechanisms of podocytes loss.

## The Anatomy and Physiology of Podocyte

Podocytes possess a complicated cellular structure, which consists of a cell body, a major process extending outwardly from their cell body, and a secondary process (foot process) surrounding the glomerular capillary loops. Major processes are restrained by microtubules and intermediate filaments, while foot processes are tethered by an actin-based cytoskeleton. Podocyte foot processes are connected by a functioning structure named slit diaphragm (SD), consisting of many proteins such as nephrin, Neph1, and podocin, which actively participate in podocyte signaling (Figure 1). Additionally, foot processes possess a thick coat (glycocalyx) with a negative charge facing the urinary space. Microtubules and intermediate filaments dominate within the cell body and the major processes, while longitudinally, bundles of microfilaments are encountered within the foot processes. Podocytes are coated by negatively charged podocalyxin, which repels each other electrostatically between the neighboring FP (Reiser and Altintas, 2016), contributing to the impairment of albumin filtration (Trimarchi, 2017) (Figure 1).

The cytoskeleton of podocytes and its interaction with GBM accounts for not only the attachment of the cells to the extracellular matrix but also the regulation of the capillary width, which is critical for determining the glomerular filtration rate (Figure 1). Many proteins of the cytoskeleton system (e.g., actin, α-actinin 4, and synaptopodin) are connected to the cytoplasmic membrane, playing roles in its relaxation and contraction, which regulate the width of the slit diaphragm and determine the size and rate of filtered molecules. Actin receives the information from CD2AP (CD2-associated protein), which translates the information from TRPC6 (Transient Receptor Potential Cation Channel Subfamily C Member 6) and from podocin, which is located in the foot process and forms part of the slit diaphragm. Podocytes are anchored to the glomerular basement membrane with anchoring proteins talin-1 and integrins. The interaction between actin, urokinase-type plasminogen activator receptor (uPAR), and integrins also determines the relaxation and contraction of podocytes (Figure 1). The SD proteins such as nephrin, Neph1, and podocin form a receptor complex; therefore, upon ligand binding, both nephrin and Neph1 become phosphorylated, recruiting adaptor molecules subsequently and coordinating junction formation and cytoskeleton dynamics (Moreno et al., 2008).

## The Cell Cycle of the Podocyte in Diseased Condition

**FIGURE 2:**
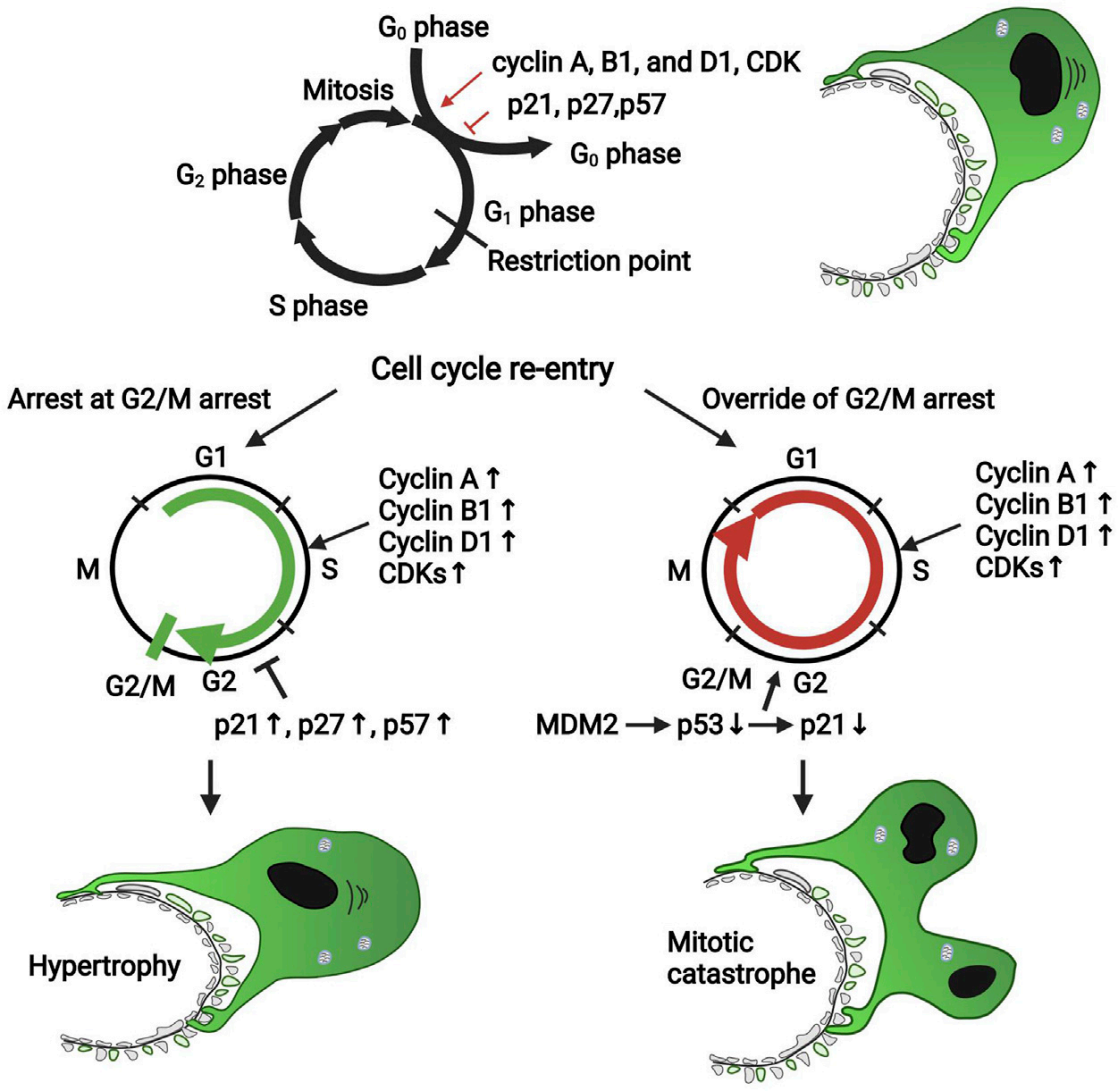
The cell cycle of the podocyte in diseased condition. Cell cycle proteins such as cyclin A, cyclin B1, cyclin D1, and CDKs promote podocyte re-entry into the cell cycle; however, the CDK inhibitors such as p21, p27, and p57 block the cell cycle. Mature podocytes express negligible cyclin A, cyclin B1, cyclin D1, and CDKs but constitutively express p27, p57, and WT-1, which maintain the cell cycle quiescence. During podocyte injury, many pathogenic factors such as FGF and TGF-β upregulated the expression of cell cycle proteins favoring the proliferation to promote the podocyte to override the G1 restriction point; also, the CDK inhibitors were upregulated to keep the cells arrest in the G2/M phase. Under some conditions, podocytes could override the G2/M restriction by MDM2 through inhibiting p53-dependent cell cycle arrest. However, due to the incomplete formation of mitosis and cytokinesis, podocytes could not complete successful division; thus, a mitotic catastrophe of podocytes occurs. CDK, cyclin-dependent kinase; MDM2, murine double minute 2.

The cell cycle is a tightly regulated event that consists of four phases: G1 phase (ready for DNA synthesis), S phase (DNA replication), G2 phase (the gap between DNA synthesis and mitosis), and M phase (mitosis and cytokinesis). Cell enters a state of quiescence at the G0 phase. The cell’s nucleus divides in the mitosis process, while the cell’s cytoplasm divides in the cytokinesis process. A balance of cyclins and cyclin-dependent kinase (CDK) (e.g., cyclin A, cyclin B1, cyclin D1, and CDK2, 4, and 6) and CDK inhibitors (p21, p27, and p57) governs the cell cycle (Figure 2). Different types of cyclins bind to different CDK at each specific phase (Shankland and Wolf, 2000), while CDK inhibitors bind to and inactivate cyclin-CDK complexes. Mature podocytes remain quiescent by constitutively expressing p27, p57, and WT-1 in the mature glomeruli. WT-1 downregulates β-catenin/Wnt signaling, which directly activates cyclin D1, controlling podocyte proliferation (Grouls et al., 2012). MDM2 negatively regulates p53 tumor suppressor, functioning as an E3 ubiquitin ligase responsible for the ubiquitination and degradation of p53 or retaining p53 in the cytosol (Iwakuma and Lozano, 2003). Multiple stimuli during podocyte injury increased the secretion of many growth factors such as angiotensin II (ANG II), fibroblast growth factors (FGFs), growth hormone (GH), and transforming growth factor-β (TGF-β), which upregulate the expression levels of cyclin A, cyclin B1, cyclin D1, and CDKs to override the G1/S transition and to drive the cell re-entering the cell cycle (Srivastava et al., 2006; Grahammer and Huber, 2016; Sakairi et al., 2010). However, in most situations, the podocytes will arrest in the G2/M phase, the exact mechanism of which is not clear. It is reported that the CDK inhibitors (p21, p27, and p57) were also increased, which prevents the cell cycle from bypassing the G1 or G2/M restriction point (Hagen et al., 2016) (Figure 2). Under some conditions, podocytes could bypass cell cycle G2/M arrest, entering mitosis by MDM2 through inactivating p53-mediated cell cycle arrest and subsequently decreasing p21 expression (Mulay et al., 2013). However, because of the incomplete formation of mitotic spindles and failure of cytokinesis due to the sophisticated anatomical structure and inadequate expression of Aurora kinase B, which is crucial for cytokinesis, aberrant mitosis (mitotic catastrophe) in podocytes takes place (Lasagni et al., 2013) (Figure 2).

## Mechanism of Podocyte Loss

Podocyte loss is a prerequisite for glomerulosclerosis; however, the underlying mechanism remains unclear. Podocyte death *in situ* on the glomerular basement membrane could result in cell detachment. Conversely, podocyte detachment leads to cell death. However, detachment of viable podocytes also occurs. In addition, we do not know whether podocytes detach because of their death or die because they lose their adhesion to GBM. Currently, in human kidney disease, podocytes injury was assessed mainly by the ultrastructural appearance of the secondary foot processes under transmission electron microscopy (EM), which are interdigitating and teethlike in normal condition but usually appear flat due to foot process effacement during podocyte injury. The assessment of podocyte death or detachment is not routinely performed in human renal biopsy specimens (Liapis et al., 2013).

**FIGURE 3:**
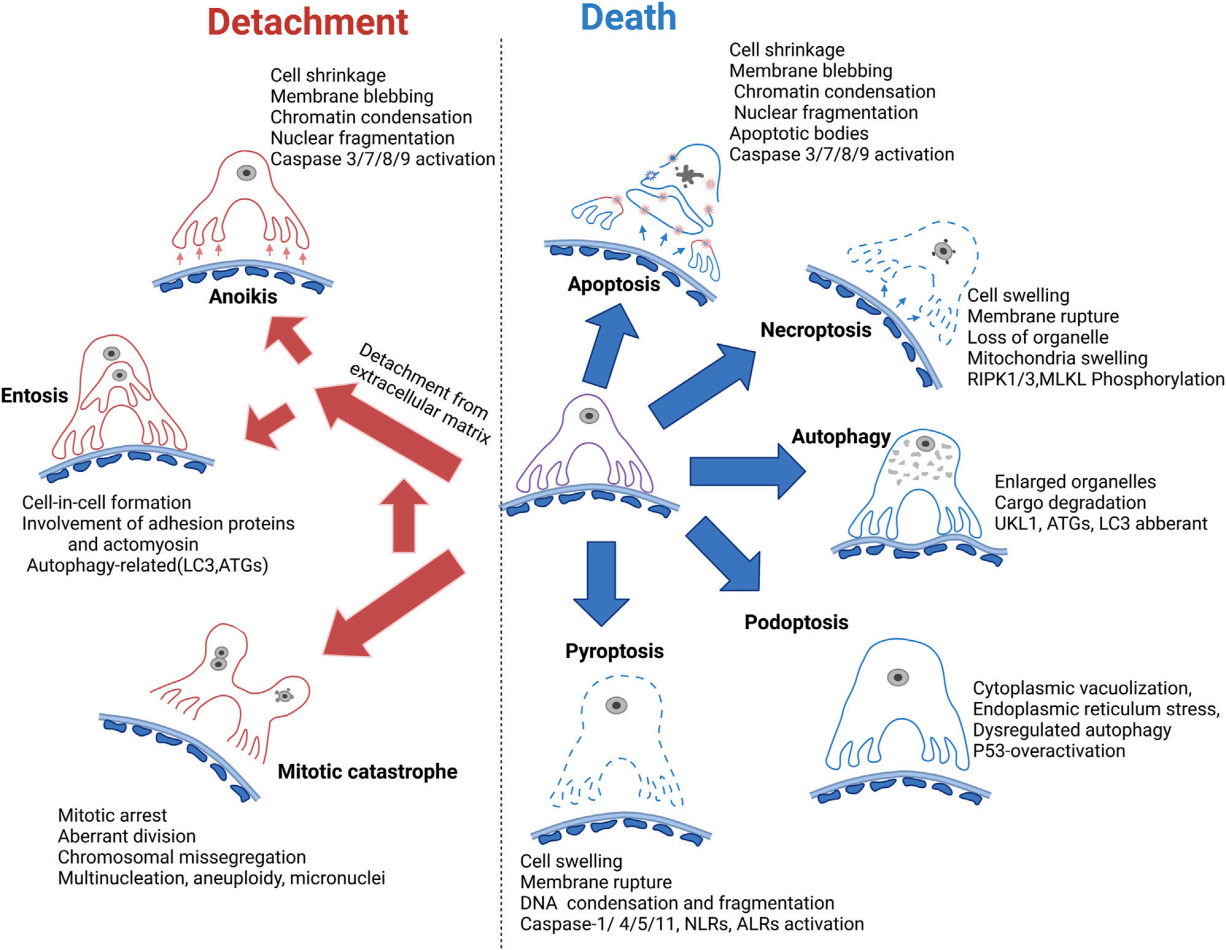
Mechanism of podocyte loss. Apoptosis is previously considered the common form of podocyte death; however, the *in vivo* evidence is lacking. Autophagy dysregulation related cell death is generally accepted as a major mechanism of podocyte cell death. Necroptosis, podoptosis, and pyroptosis are also involved in podocyte death. Meanwhile, podocyte detachment and detachment-induced cell death such as anoikis and entosis remain the major mechanism of podocyte loss. Mitotic catastrophe could promote podocyte detachment from GBM. GBM, glomerular basement membrane. RIPK, receptor-interacting protein kinase 1; MLKL, mixed lineage kinase domain-like pseudokinase; UKL1, unc-51 like autophagy activating kinase 1; LC3, microtubule-associated protein light chain 3; ATGs, autophagy-related proteins; NLRs, NOD-like receptors; ALRs, AIM-2-like receptors.

There are numerous mechanisms for podocyte loss (Figure 3), including detachment related cell death (anoikis and entosis), programmed apoptotic cell death (apoptosis), programmed nonapoptotic cell death (e.g., autophagy and podoptosis), immune-reactive cell death such as pyroptosis, and other types of cell death such as necroptosis and mitotic catastrophe-induced cell death. Each individual type of cell death is discussed separately.

## Podocyte Loss due to Cell Death

### Apoptosis

Apoptosis is the process of programmed cell death, the first sign of which is the condensation of the nuclear material, accompanied by cell shrinkage and the formation of many membrane-bound, ultra-structurally well-preserved fragments resulting from cell breakup (Westman et al., 2019; Yan et al., 2020). Both the extrinsic and intrinsic apoptotic pathway share the common executioner caspases, mainly caspase 3, and ultimately promote chromatin condensation, nuclear fragmentation, and formation of apoptotic bodies (Galluzzi et al., 2018). Usually, terminal deoxynucleotidyl transferase dUTP nick-end labeling (TUNEL) assay using live cell-impermeant staining in combination with annexin V conjugates and caspase assay such as cleaved caspase-3 was used to assess apoptosis (Yan et al., 2020).

Apoptosis has been previously considered as a common cause of podocyte loss because podocytes apoptosis was observed *in situ* in various experimental animal models such as diabetic nephropathy, progressive glomerular sclerosis, and transforming growth factor-β1 (TGF-β1) transgenic mice (Schiffer et al., 2001; Susztak and Böttinger, 2006; Jia et al., 2008). Nevertheless, the data favoring podocyte apoptosis have been generated mostly from *in vitro* studies, and evidence derived from *in vivo* experiments has been mainly based on tissue staining with cleaved caspase 3 or TUNEL (Schiffer et al., 2001; Ryu et al., 2011). It is known that detecting DNA fragmentation *in situ via* TUNEL as evidence of apoptosis has been challenged (Graslkraupp, 1995) because TUNEL detects different kinds of cell death such as necrosis and autolytic cell death. The presence of apoptotic bodies in podocytes by EM is the most reliable indicator of apoptosis; however, no detection of apoptotic bodies has been reported in human kidney biopsies by EM so far (Nagata, 2016). It is important to understand that the time course of apoptosis is very fast (Tharaux and Huber, 2012; Westman et al., 2019), irrespective of the initiating insult. Usually, phagocytes eliminate apoptotic bodies within hours (Matter, 1979); thus, the chance of observing the apoptotic cells is very low. Even if the phagocytosis is absent, apoptotic cells can detach rapidly or become secondary necrotic cells, which is difficult to distinguish from primary necrosis. Therefore, it is difficult to confirm the role of podocyte apoptosis in kidney disease (Liapis et al., 2013).

### Autophagic Cell Death

Autophagic cell death (ACD) is a type of nonapoptotic cell death, which occurs in the absence of chromatin condensation but through a lysosome-dependent degradation pathway, accompanied by large-scale autophagic vacuolization of the cytoplasm (Galluzzi et al., 2018). The detection methods include measurement of autophagic activity (e.g., autophagic flux) and indirect analysis with autophagy-specific key molecules, such as UKL1 (unc-51 Like Autophagy Activating Kinase 1), LC3 (microtubule-associated protein light chain 3), and ATG (autophagy-related proteins) (Yan et al., 2020).

Autophagy is extraordinarily important in maintaining the homeostasis of terminally differentiated cells such as podocytes under physiological and stress conditions (Asanuma et al., 2003). Autophagy deficiency in podocytes results in the loss of cellular protein homeostasis and a dramatic acceleration of glomerular diseases (Hartleben et al., 2010). In diabetic patients and animal models with massive proteinuria, podocyte autophagy was observed to be insufficiently accompanied by podocyte loss (Yasuda-Yamahara et al., 2015). The transgenic mice with autophagy-specific deficiency in podocytes developed massive proteinuria and podocyte loss in a high-fat diet (HFD)-induced diabetic model, in which minimal proteinuria was often induced (Tagawa et al., 2016). Serum from diabetic patients and experimental rodents with massive proteinuria could cause autophagy insufficiency, lysosome dysfunction, and apoptosis of cultured podocytes (Yasuda-Yamahara et al., 2015). This indicates that autophagy impairment in podocytes under diabetic conditions plays an important role in the pathogenesis of podocyte loss and massive proteinuria in diabetic nephropathy (Yasuda-Yamahara et al., 2015; Tagawa et al., 2016). It has been shown that activation of autophagy suppresses podocyte apoptosis and glomerulopathy in the adriamycin-induce mice model; conversely, inhibition of autophagy promoted adriamycin-induced podocyte injury *in vitro* and *in vivo* (Yi et al., 2017). All of these findings suggest the dysregulated autophagy-related podocyte death both *in vivo* and *in vitro*.

mTOR (mammalian target of rapamycin) pathway is the best-characterized repressor of autophagy (Laplante and Sabatini, 2012). The mechanistic target of rapamycin complex 1 (MTORC1) is one of the multiprotein complexes of mTOR, inhibiting the autophagy process by phosphorylating ULK1 (Ganley et al., 2009). There are pieces of evidence showing that MTORC1 activation was strengthened in podocytes of individuals with progressive diabetic nephropathy, and abnormal MTORC1 activation recapitulated many diabetic kidney disease features in nondiabetic rodents such as loss of podocyte, thickening of the glomerular basement membrane, glomerular mesangial expansion, and proteinuria (Inoki et al., 2011). Rapamycin, the selective inhibitor of mTOR protein kinase, was proved to reduce podocyte apoptosis by inhibiting the mTOR/P70S6K/4EBP1 signaling pathway and activating podocyte autophagy in idiopathic membranous nephropathy (IMN) mice model (Wu et al., 2013). AGEs can promote the activation of mTOR in human podocytes and inhibit nuclear translocation of the transcription factor EB, downregulating the expression of autophagy-related genes, thereby inhibiting the formation of autophagosomes in the cell and simultaneously increasing the early and late apoptosis of podocytes.

Altogether, these data suggest that autophagy dysregulation related cell death could be a major pathway for podocyte death, particularly in diabetic kidney disease.

### Necroptosis

Necroptosis is a regulated cell death that shows the same characteristics as necrosis in morphology (lysis of cell membrane) and is different from the apoptosis signal pathway in its mechanism. A variety of molecules such as RIPK1 (receptor-interacting protein kinase 1), RIPK3 (receptor-interacting protein kinase 3) and its substrate MLKL (mixed lineage kinase domain-like pseudokinase) participate in the regulation of cell necroptosis (Galluzzi et al., 2018). Western blot assay of the marker involved in the necroptosis pathway, mitochondrial potential analysis by fluorescent probes, and morphology observation by EM can be applied to the assessment of necroptosis (Yan et al., 2020).

The studies on podocyte necroptosis are rare. A few studies suggested that high glucose could induce necroptosis in podocytes, which is regulated by ubiquitin C-terminal hydrolase L1 (UCH-L1) *via* RIPK1/RIPK3 pathway, evidenced by western blot assay of necrosis pathway markers such as RIPK3 and MLKL and scanning EM (Xu et al., 2019). Sosna and co-workers showed that HtrA2/Omi (a serine protease previously implicated in programmed cell death) and ubiquitin C-terminal hydrolase L1 (UCH-L1) regulate TNF-induced necroptosis in podocytes using necroptosis specific inhibitors such as necrostatin-1 (Sosna et al., 2013). Recently, Guo and co-workers observed that the activation of RIPK3 and MLKL was observed in kidney podocytes from class IV human lupus nephritis and experimental animal models such as lupus-prone NZM2328 and MRL/lpr mice, as evidenced by staining of phosphorylated forms of RIP3 and MLKL in podocytes. Furthermore, GSK872 (a specific RIPK3 kinase inhibitor) treatment alleviates the disease activity of MRL/lpr mice (Guo et al., 2019). Thus, these limited studies suggest that podocyte necroptosis might occur in diseased kidneys.

### Pyroptosis

Pyroptosis is an inflammatory form of programmed cell death regulating immunity response (Xiong et al., 2020). There are two pathways related to pyroptosis that have been identified: the canonical pathway mediated by caspase-1 and induced by inflammatory bodies such as NLRP3 (NLR family pyrin domain containing 3) and the noncanonical pathway mediated by caspase-4/5/11 and activated by lipopolysaccharide (LPS) (Yu et al., 2021; Zhang et al., 2021). The common consequence of the above two pathways is the cleavage of gasdermin-D (GSDMD) into N-terminal fragments of GSDMD (GSDMD-NT), which played an essential role in the pore formation through insertion into the cell membrane and subsequent cellular contents release such as IL-1β and IL-18 (Evavold et al., 2021; Yu et al., 2021).

The activation of NLRP3 inflammasomes could recruit caspase-1 *via* the adaptor protein of CARD-PYRIN (von Moltke et al., 2013) and induce pyroptosis subsequently. Murine podocytes expressed three essential components of the NLRP3 inflammasome complex (Zhang et al., 2012). NLRP3 inflammasome activation played an important in initiating obesity-associated podocyte injury (Boini et al., 2014) and promoting diabetic nephropathy development in the diabetic rodents (Shahzad et al., 2015). The expression levels of proteins involved in the pyroptosis pathway such as GSDMD-N, NLRP3, cleaved caspase-1, and IL-1β were considerably enhanced in the mouse models treated with a high-fat diet or streptozotocin and in the high glucose-treated podocytes (An et al., 2020; Li et al., 2020; Zhan et al., 2020). Activation of NLRP3 inflammasome could be induced by high glucose and blockade of its activation or inhibition of caspase-1 reduced the production of IL-1β and mitigated podocyte and glomerular injury in diabetic mouse models (Gao et al., 2014; Shahzad et al., 2015). In addition, NLRP3 inflammasomes are also activated in podocytes from patients with lupus nephritis and lupus-prone mice (Fu et al., 2017). The activation of NLRP3 inflammasome and caspase-1, as well as increased IL-1β production, also plays a crucial role in podocyte injury induced by hyper-homocysteinemia (hHcys) (Abais et al., 2014; Xia et al., 2014).

Recently, the evidence demonstrated that noncanonical pyroptosis-regulated cell death was also involved in the pathogenesis of diabetic nephropathy and disease progression. They showed that the expression levels of caspase-11 and GSDMD-N in podocytes were significantly increased, associated with the decreased expression level of podocyte-specific proteins such as nephrin and podocin, effacement of podocyte foot processes, expansion of glomerular matrix, and higher urinary albumin-to-creatinine ratio. These data suggest a role of pyroptosis in podocyte injury in diabetic nephropathy (Cheng et al., 2021). However, evidence of podocyte pyroptosis in human kidney disease is still lacking.

### Podoptosis

Podoptosis is referred to as p53 overactivation-related cell death (Thomasova et al., 2015). It is known that the maintenance of p53 balance is crucial for podocyte survival; thus, many proteins enriched in podocytes such as WT-1 (Maheswaran et al., 1995), MDM2 (Mulay et al., 2013), and RARRES1 (Chen et al., 2020) interact with the p53 pathway. MDM2 could promote podocyte loss by overriding cell cycle G2/M restriction and entering mitosis through degradation of p53 or by retaining p53 in the cytosol (Mulay et al., 2013; Tang et al., 2017). It also prevents p53 overactivation-related cell death (Thomasova et al., 2015). Morphologically, podoptosis is characterized by massive cytoplasmic vacuolization and signs of endoplasmic reticulum stress. Mechanistically, the p53-overactivation-related cell death is p53 inhibitor-sensitive but pan-caspase inhibitor-insensitive, suggesting that it does not require caspase activation, and therefore this p53 overactivation-related cell death is not apoptosis. Moreover, podoptosis is not affected by specific inhibitors for pyroptosis, pyronecrosis, necroptosis, ferroptosis, and parthanatos. Thus, podoptosis, a p53 overactivation-related podocyte death, is a new form of cell death (Thomasova et al., 2015).

### Detachment-Induced Podocyte Loss

Many studies confirmed the presence of viable urinary podocytes in human and experimental glomerular diseases (Vogelmann et al., 2003). These urine-derived podocytes from animal models and human subjects have been cultured and made into immortalized cell lines (Petermann et al., 2003; Sakairi et al., 2010). Urinary podocytes from patients with active glomerular disease shared the same morphology and growth pattern with primary podocytes isolated from human kidneys. However, the cultured urinary podocytes from healthy control individuals showed very limited growth capability and underwent death much sooner than those of patients with glomerular disease. These results suggest that viable podocytes may detach from the glomerular tuft of diseased kidneys due to the local altered microenvironment instead of the defect of podocyte per se in patients, whereas urine podocytes in healthy individuals are most likely from the shedding of senescent cells.

There is very limited insight into the mechanisms that mediate the detachment of podocytes. Currently, podocyte detachment is thought to be caused by the increased mechanical shear forces and/or disruption of the cell-GBM adhesion (Kriz and Lemley, 2015). Glomerular hypertension and hyperfiltration increase the shear stresses on podocytes, promoting the detachment of podocytes (Benzing and Salant, 2021). Podocyte-GBM adhesion is modulated by several integrins and cytoskeletal protein such as αVβ3 or α3β1 integrin and talin-1 (Nagata, 2016), disruption of which causes podocyte detachment (Yu et al., 2013; Tian et al., 2014). It is reported that the significant decrease of α3β1 integrin at the basal plasma membrane of podocytes takes place as early as one month after the occurrence of hyperglycemia, at the time when the morphological changes of glomerular basement membrane are not yet established; thus, it might be an early event which promotes podocyte detachment before the onset of diabetic nephropathy (Regoli and Bendayan, 1997). Podocytes are especially susceptible to injury in many glomerular diseases, during which the cell shapes of podocytes change, including retraction and broadening of their foot processes. There are two opposite hypotheses for this observation: this could be an early sign of podocyte detachment or this could be a defense mechanism of podocytes against detachment from the GBM (Kriz and Lemley, 2015). On the one hand, podocyte effacement reduced the buttressing force of podocytes and the compressive force of the GBM, subsequently drastically increasing capillary pressure and local fluid flow at the barrier due to the decreased filtration area, thus leading to loss of podocytes through detachment because of enhanced transverse shear stress (Benzing and Salant, 2021). On the other hand, podocyte effacement closes the slits and could prevent podocytes from suffering from high flows and shear stresses (Kriz and Lemley, 2015).

Under high glucose conditions, podocytes undergo cell hypertrophy to compensate for podocyte loss (Hoshi et al., 2002). However, hypertrophy of podocytes could result in the alteration of structure that leads to the podocyte detachment from the GBM (Kriz et al., 2014). Complement activation in lupus nephritis can impede podocyte focal adhesions (Topham et al., 1999); meanwhile, exposure to the circulating factors in focal segmental glomerulosclerosis (FSGS) might dysregulate the actin cytoskeleton (Kemeny et al., 1995). The alteration of focal adhesions and actin cytoskeleton leads to podocyte detachment. Consistently, podocytes with CD2AP- deficiency are more susceptible to cell death induced by detachment than wild-type podocytes (Huber et al., 2003). mTORC1 activation in diabetic conditions may induce epithelial-mesenchymal transition-like phenotypic changes in podocytes, promoting podocyte detachment from GBM (Inoki et al., 2011). Altogether, these studies support the early occurrence of podocyte detachment as a major mechanism of podocyte loss.

### Mitotic Catastrophe-Induced Podocyte Detachment

Mitotic catastrophe is a sequence of events resulting from aberrant mitosis caused by DNA damage or any other aberrations affecting the mitotic process (Galluzzi et al., 2018). Mitotic catastrophe is morphologically characterized by nuclear abnormalities, including multinucleation (aneuploidy), micronuclei, or irregularly shaped nuclei (Galluzzi et al., 2018). The cells suffering from mitotic catastrophe often undergo programmed cell death, such as intrinsic apoptosis.

Mature podocytes sustained cell cycle quiescence. Differentiated podocytes could re-enter the cell cycle in response to various stimuli; however, they cannot accomplish cytokinesis despite karyokinesis, thus resulting in mitotic catastrophe (Liapis et al., 2013). Recently, Hara and co-workers examined urinary podocytes, 53.5% of which were considered as mitotic catastrophe cells, with the character of abnormal shape, aneuploidy, mitotic spindles, and denucleation (Hara et al., 2019). They suggest that in diabetic patients, the majority of urinary podocytes show the character of mitotic catastrophe, not apoptosis. Mitotic catastrophe has been observed in a variety of nephropathies in which podocytes were extensively damaged, such as membranous nephropathy, focal segmental glomerulosclerosis (FSGS), diabetic kidney disease, and IgA nephropathy (Vakifahmetoglu et al., 2008; Liapis et al., 2013). Consistent with the urinary analysis, the detection of mitotic podocytes in human glomerular disease from IgA nephropathy, collapsing FSGS, and drug-induced minimal change disease were all observed in the urinary space away from the glomerular tuft (Liapis et al., 2013). Blockade of MDM2 (an inhibitor of p53-dependent cell cycle arrest) prevented podocytes from aberrant mitosis by inducing G2/M arrest and detachment of dying aneuploid podocytes in the adriamycin nephropathy mice model (Mulay et al., 2013), supporting the hypothesis that mitotic catastrophe plays important roles in podocyte detachment. The mitotic podocytes detach from the GBM mainly because of the inability of actin assembly to form mitotic spindle and support the cytoskeletal structure of foot processes at the same time (Lasagni et al., 2013). These shreds of evidence suggest that mitotic catastrophe plays an important role in podocyte detachment.

### Detachment-Induced Podocyte Death

Currently, the detachment of epithelial cells from the extracellular matrix could trigger two kinds of cell death, anoikis and entosis. Anoikis is a kind of apoptosis induced by the loss of attachment to the extracellular matrix (ECM) or the neighboring cells, which could be regulated *via* both the intrinsic and extrinsic apoptotic pathways (Frisch and Francis, 1994; Cardone et al., 1997; Reginato et al., 2003). Gilmore and co-workers demonstrated that cells lacking ECM attachment suffered from classical apoptosis eventually (Gilmore, 2005). However, the mechanism underlying the relationship between cell architecture alteration and apoptosis needs to be further explored. Overholtzer and co-workers found that detached epithelial cells from the extracellular matrix and subsequent loss of integrin signaling would trigger entosis, which is a nonapoptotic cell death pathway, during the process of which one cell invades or is engulfed by another cell (Overholtzer et al., 2007). However, there is no specific method to detect entosis currently. The detection of the typical cell-in-cell structure by fluorescence imaging and EM was applied to identify the cells with entosis (Yan et al., 2020). However, this has not been reported in human kidney disease.

### Podocyte Shedding of Viable Cells in the Urine

Despite the above observations that favor the early and essential role of detachment in podocyte loss, there are two remaining issues regarding the urinary podocytes: 1) the identification of podocytes mainly relied on the podocalyxin-positive cells. Nevertheless, podocalyxin-positive cells are not necessarily podocytes since many other cells express podocalyxin, such as hematopoietic stem cells, vascular endothelial cells, platelets, and macrophages (Orikasa et al., 1996). 2) Though there is evidence that urinary podocytes can be cultured, these cells might be from other sources. For instance, podocyte progenitor cell populations such as CD133 and CD24 double-positive stem cells and the cells of renin lineage, identified as a FoxD1þ stromal progenitor (Nagata, 2016), might contribute to these observations. The co-expression of WT1 and α-smooth muscle actin or cytokeratin 8 might indicate this possibility, although co-expression of these markers could indicate less differentiated podocytes (Vogelmann et al., 2003).

### Podocyte Domino Effect

The “podocyte domino effect” is recently identified as a novel mechanism of progressive podocyte loss (Mulay et al., 2013; Nagata, 2016). Podocyte damage due to primary insult can spread injury by inducing secondary damage to the remaining intact podocytes, even though the initial insult is very short-lived (Nagata, 2016). This was suggested by utilizing chimeric mice, in which only a subpopulation of podocytes expressed hCD25, the receptor for the immunotoxin LMB2. LMB2 injection to the chimeric mice initially causes the podocyte injury limited to the subpopulation of podocyte expressing hCD25; however, hCD25-negative podocytes developed injury as early as the four days after the LMB2 injection, evidenced by foot process effacement and decreased expression of podocyte markers such as nephrin, podocin, and podocalyxin (Matsusaka et al., 2011). The exact mechanism of this observation remains to be determined. However, this could be due to podocytes-podocytes crosstalk or podocytes-endothelial cells-podocytes crosstalk. When podocytes are injured, they secrete cytokines that could damage neighborhood podocytes or glomerular endothelial cells, which in turn affect other podocyte functions.

## Conclusion

Podocyte number was correlated with the amount of proteinuria and extent of glomerulosclerosis. Due to its limited regeneration capacity, it is important to understand the underlying mechanism of podocyte loss, and therefore, we could develop effective measures to prevent their loss. Several common cell death pathways likely apply to podocyte loss in kidney diseases; however, podocytes may have unique pathways such as anoikis, podoptosis, and mitotic catastrophe. Podocytes may undergo cell death and detachment sequentially; however, the evidence of increased viable urinary podocytes in patients with glomerular disease supports that they may detach and stay alive. The dead podocytes are eliminated quickly by phagocytosis, so it is difficult to detect them *in vivo* and therefore may mask the fact that they are dead before detachment. Our understanding of the mechanisms of podocyte loss needs to be improved with more studies. Ultimately, we could develop effective measures to prevent podocyte loss as a therapy for glomerular disease.
